# Psychological Predictors of Seeking Help from Mental Health Practitioners among a Large Sample of Polish Young Adults

**DOI:** 10.3390/ijerph13111049

**Published:** 2016-10-26

**Authors:** Lidia Perenc, Mieczyslaw Radochonski

**Affiliations:** 1 Medical Faculty, University of Rzeszow, Rzeszow 35-310, Poland; lidiaiadam.perenc@wp.pl; 2 Faculty of Education, University of Rzeszow, Rzeszow 35-310, Poland

**Keywords:** attitudes, help-seeking, mental health, young adults, prevention

## Abstract

Although the corresponding literature contains a substantial number of studies on the relationship between psychological factors and attitude towards seeking professional psychological help, the role of some determinants remains unexplored, especially among Polish young adults. The present study investigated diversity among a large cohort of Polish university students related to attitudes towards help-seeking and the regulative roles of gender, level of university education, health locus of control and sense of coherence. The total sample comprised 1706 participants who completed the following measures: Attitude Toward Seeking Professional Psychological Help Scale-SF, Multidimensional Health Locus of Control Scale, and Orientation to Life Questionnaire (SOC-29). They were recruited from various university faculties and courses by means of random selection. The findings revealed that, among socio-demographic variables, female gender moderately and graduate of university study strongly predict attitude towards seeking help. Internal locus of control and all domains of sense of coherence are significantly correlated with the scores related to the help-seeking attitude. Attitudes toward psychological help-seeking are significantly related to female gender, graduate university education, internal health locus of control and sense of coherence. Further research must be performed in Poland in order to validate these results in different age and social groups.

## 1. Introduction

According to current estimates, the number of mental health disorders in Poland has been apparently rising over the last two decades or so [1,2]. One of the major causes of this state of affairs is related to the consequences of far-reaching socio-economic changes that occurred in this country during last 25 years. It seems that the existing forms of mental health care have become largely inadequate, and—therefore—the present mental disorder prevention system requires substantial transformation. Despite the high prevalence of mental health problems among Polish adolescents and young adults, only a small proportion may be said to receive any professional help. Significantly, mental health care centres are located mostly in a bigger cities and towns, and this results in restricted access for people living in rural areas. Obviously, better access to appropriate mental health care would positively influence their future, and this is the reason why those individuals who face urgent mental health problems need early intervention and professional consultation.

In order to develop an effective therapeutic interventions, there are several important factors that need to be taken into consideration. One of them is the general readiness of young people to seek help from mental health professionals. Former studies on help-seeking attitudes and behaviours tended to focus on demographic variables such as gender, age, socio-economic status, race and previous experience with mental health interventions [3,4], and one may conclude that these studies established some well-defined relationships between certain factors such as gender [5], educational level [6], family network [7] and others. Although the authors cited above have all contributed significantly by providing useful knowledge on some vital factors that account for help-seeking attitudes, some important psychological variables have remained either merely touched upon or utterly unexplored and unaccounted for. Presumably, certain psychological variables, such as health locus of control and sense of coherence, seem to play an important role in determining help-seeking behaviours. The concept of health locus of control is based on the general assumption that management of health issues resides either within the person or powerful others. Individuals with internal health locus of control are expected to demonstrate positive attitudes towards seeking help from mental health practitioners. Literature on the subject shows that some of the studies carried out earlier confirmed this general assumption [8,9].

Personality traits and individual differences also belong to the body of important factors that influence one’s attitude to seek mental health services. In their study on the relationship between help-seeking and personality traits Barwick, de Man and McKelvie [10] found that those persons who are characterized by high trait-anxiety, low self-esteem and an external locus of control more frequently assumed a negative attitude toward seeking professional help. Another personality trait that affects help-seeking behaviour is openness to experience which may be defined as the disposition to adjust personal beliefs and behaviour when a given individual is exposed either to new information or new ideas [11]. Those who are open to new experiences usually tend to exhibit more positive attitude toward seeking professional psychological help because it fulfils their need for curiosity, for example, participation in psychological therapy [12,13].

Another personality variable, truly vital from point of view of the goals set to this study, is sense of coherence (SOC). According to the accepted definition, it is a pervasive, dynamic and enduring feeling of confidence that life and the surrounding environment are predictable and there is a high probability that things will work out according to reasonable expectations [14]. The concept of SOC comprises three components, that is comprehensibility, manageability and meaningfulness. Like other personality domains, the SOC can motivate individuals to use personal and environmental resources in order to achieve optimal functioning. It is assumed that a higher level of the SOC may be related to positive attitude toward seeking professional psychological help. As such, the SOC should be taken into consideration in the studies aimed at analyzing the relationship between this variable and help-seeking behaviour.

Interesting changes in attitudes toward seeking mental health services over time were observed by Currin et al. [15] in their study carried out on groups of older and younger adults in 1977, 1991 and 2001. The results were rather ambiguous as they found increasingly positive attitudes among the older group from 1977 to 1991, and both the older and younger participants demonstrated more negative help-seeking attitudes from 1991 to 2001. In contrast to this finding, Mojtabai [16] revealed a marked improvement in help-seeking attitudes over the decade between 1992 and 2003, both in relation to psychotherapy and drug treatment, so these studies present some conflicting evidences. One possible explanation for this state of affairs may have to do with the measurement reliability and differences between those samples that were studied.

The aim of the present study is to investigate the relationship between selected demographic and psychological variables and attitude towards seeking professional psychological help in a large sample of the Polish young adults. To the best of our knowledge, no research on the relationship between these variables was carried out in Poland on this scale thus far yet. Note that the results of this study also can be used for comparison with results of research performed in different cultural settings.

## 2. Materials and Methods

### 2.1. Study Design

This cross-sectional survey was carried out in November and December 2015. It utilizes non-experimental design with one dependent variable (attitude toward seeking help from mental health practitioners) and five independent variables (health locus of control, sense of coherence, gender, level of university education and social background).

### 2.2. Participants

This survey included a large, randomly selected, sample of 1706 students from University of Rzeszow (Rzeszow, Poland). The university is the largest higher educational institution located in south-east Poland, with an enrolment of 17,221 students during the academic year 2015/16. Due to its location it attracts students from diverse socioeconomic backgrounds (see Table 1).

The study group comprised only residential students who formed the young adult population of this survey. Their age ranged between 19 and 26 years (mean 22.4). As shown in Table 1, the study included 622 males (36.46%) and 1084 females (63.54%). According to education parameter, 943 (55.27%) were students of undergraduate courses (licentiate), and 763 (44.73%) were enrolled in graduate courses (master degree). In terms of their social milieu, 715 (41.91%) participants originated from urban background and 991 (58.09%) were from the country. Over 95% of them declared their religious affiliation as Roman Catholic, and the rest were either agnostic or atheist. 

### 2.3. Instruments

The study used a method of diagnostic survey. It comprises the following tools structured in the Polish language:

Demographic sheet: The sheet required the participants to provide their gender, age, social background, level and type of university course, and religious involvement. Additionally, they were expected to declare whether they had previously been subject of psychological therapy or counselling.

Attitude Toward Seeking Professional Psychological Help Scale-Short Form (ATSPPHS): This scale is meant to measure one’s willingness to seek help from mental health practitioners in situations when a serious psychological problems are encountered. It consists of 10 items chosen from the original 29-item version developed originally by Fischer and Turner [17], and later transformed into a one-dimensional, 10-item scale [18]. The items are responded on a 4-point Likert-type scale ranging from “strongly disagree” (1) to “strongly agree” (4). Higher scores indicate more positive attitudes toward seeking assistance from mental health practitioners in solving personal or family problems. Fischer and Farina [18] obtained an internal coefficient alpha of 0.84 and a test-retest reliability of 0.80. Also, a construct validity for this instrument was demonstrated by significant correlations between individual scores and their scores on an independent measure of help-seeking behaviour [18].

Multidimensional Health Locus of Control Scale (MHLC): The Polish version of this scale contains 18 items concerning health issues with which the responding person could either agree or disagree. The response pattern is on a 6-point Likert-like scale ranging from “strongly disagree” (1) to “strongly agree” (6). Depending on the response to every statement, the appropriate number of points is individually attributed. The instrument consists of three subscales each of which contains six items. The total score for each of the subscales ranges from 6 to 36. The emerging tendency shows that the higher the score, the stronger the belief that a given factor has an important influence on health. The results obtained in the scale were calculated, according to the diagnostic key, separately for the following dimensions of health locus of control:

- *internal control* (I)—the respondent believes that the control over his health depends on him,
- *impact of others* (O)—the respondent is convinced that his own health is influenced by others, mainly the health care workers,
- *chance* (C)—the respondent believes that external factors, being out of his control, decide about his health condition.

Juczynski [19] reported satisfactory psychometric traits for the Polish version of MHLC (Cronbach alpha of 0.74 for internal control, 0.54 for impact of others and 0.69 for chance subscales).

Orientation to Life Questionnaire (SOC-29): This instrument is designed to measure sense of coherence, a relatively stable orientation towards stressful life events. The Polish version of the scale contains 29 items related to the following elements of the sense of coherence: comprehensibility (i.e., a sense that the world is rational, understandable, consistent and predictable), manageability (i.e., a sense of accessibility of resources allowing to cope with life situations), and meaningfulness (i.e., a sense that things are worth making commitments) [20]. The response format is on a 7-point Likert scale (always/often to never). Some items are scored in a reverse order. Higher scores indicate a higher sense of coherence while low scores mean low sense of coherence.

### 2.4. Procedure

Prior to data collection, this study was carefully reviewed and approved by the Bioethical Committee of the University of Rzeszow. The young adults were recruited from different university faculties and courses by means of random selection. Those students who belonged to the selected courses were asked to stay in the teaching room at the end of the lectures. Following their expressed consent and willingness to participate in the survey, they were asked to complete the questionnaires anonymously and independently, and the completion of the instruments took approximately 15–20 min. Data collection took a period of two months. The young adults participating in the survey were in no way compensated for their participation.

## 3. Results

Basic statistical techniques, namely mean, standard deviation, *t*-test for independent samples and Pearson r coefficient of correlation were used in this survey. A *t*-test was performed to explore possible differences among participants in attitude toward seeking professional psychological help based on gender, educational level, social background and psychological factors. Pearson r correlation analysis was conducted in order to find out what the direction and magnitude of the relationship between the variables are. This applies to all psychological dimensions that feature in this study, including sense of coherence and health locus of control. The resulting data-set was subjected to analysis using the STATISTICA 9.0 software (Dell Software Inc., Austin, TX, USA). The significance level of *p* < 0.05 was assumed to indicate statistically significant differences and correlations.

According to Fischer and Farina [18] the ATSPPH scale displayed the ability to differentiate between college students who choose to seek or fail to choose to seek professional psychological help. Higher scores meant that a respondents had a positive attitude toward this type of behaviour. In our study female participants had significantly higher scores than male participants (24.85 vs. 21.12, *p* < 0.01) (Table 2). Although the young adults’ average responses to the ATSPPHS items corresponded with a “higher” responsive option, the male respondents apparently have demonstrated more uncertainty about their willingness to seek assistance from mental health professionals. Also level of university education had a significant impact on help-seeking attitude because the graduate students achieved much higher results than their undergraduate colleagues (25.70 vs. 20.46, *p* < 0.05). Likewise, there was no significant difference in mean scores between participants regarding their social background. Both participants from urban and rural areas expressed similar intensity of attitudes toward seeking professional help in solving their psychological problems.

Table 3 is meant to summarise the findings of the research on health locus of control among the studied young adults. The analysis has revealed that the mean value of *internal* health locus of control in males was 28.72 and exceeded that of females which amounted to 25.36. In this case, the difference was statistically significant at the 0.05 level. The mean value in the *influence of others* subscale for males (17.30) was significantly less than in case of females (21.84; *p* < 0.01). No statistically significant difference was found between results of males (18.45) and females (17.26) in the *chance* subscale. 

It was also observed that the mean value of *internal* health locus of control among participants studying at the graduate level was 29.72 points, while in the group of undergraduate students it amounted to 23.60 points. Also in this case the difference was statistically significant (*p* < 0.01). An opposite direction of difference between the compared groups was obtained in results related to the *chance* subscale. The participants studying at undergraduate level obtained 19.85 points, while those from graduate studies achieved only 15.43 points (*p* < 0.05). In relation to the influence of others subscale, there was not statistically significant difference (*p* > 0.05).

The social background of the participants is another variable that influences difference in the MHLC scale scores. Young adults from urban area obtained significantly higher *p* < 0.01) scores in *internal* health locus of control subscale than those from rural area (27.60 vs. 20.82). Much smaller difference between the compared groups has appeared in the *influence of others* subscale. The results of the participants from rural area (21.45) exceeded that from urban area (18.10) (*p* < 0.05). In relation to the *chance* subscale, the scores obtained by urban and rural participants were not significantly different (*p* > 0.05).

As it is shown in Table 4, male participants had a significantly higher global SOC score compared to female individuals (139.32 vs. 131.11, *p* < 0.05). Moreover, the role of gender as a determinant of other components of SOC was significant in relation to comprehensibility (47.59 vs. 43.21, *p* < 0.05) and manageability (47.42 vs. 41.85, *p* < 0.01). The effect of the gender factor on meaningfulness was insignificant (44.31 vs. 45.05, *p* > 0.05). The effect of educational level on the SOC domains was differentiated. Participants studying at graduate level had significantly higher scores in the following domains: meaningfulness (42.14 vs. 46.82, *p* < 0.01) and comprehensibility (43.71 vs. 47.44, *p* < 0.05), whereas the difference in manageability was not significant (*p* > 0.05). The effect of educational level on the global SOC score was significant (*p* < 0.05). Interestingly enough, social background of the participants had marginal effect on the global SOC results. Only in the domain of manageability the participants from rural area had higher scores than those from urban background (42.67 vs. 48.35, *p* < 0.01), and this was one of the unexpected findings of this study.

The correlation analysis results show unambiguously that two of the sociodemographic variables and five of the psychological variables are significantly related to young adults’ attitudes toward seeking professional psychological help (Table 5). The findings of this study revealed moderate relationship between female gender and attitude toward seeking help from mental health professionals (r = 0.345, *p* < 0.05). Regarding level of university education, the ATSPPH scores were relatively strongly related to the graduate studies (r = 0.512, *p* < 0.01). Among psychological variables, the strongest correlation turned out to obtain between internal health locus of control and attitude toward seeking professional psychological help (r = 0.536, *p* < 0.01). This suggests that the more a given individual perceives health related to a personal responsibility, the more favourable disposition to seek professional psychological help he/she displays. Additionally, all domains of sense of coherence correlated significantly with the ATSPPH scores. The highest correlation coefficient showed the comprehensibility domain (r = 0.473, *p* < 0.01). In case of the manageability and meaningfulness domains the correlations fell within moderate range (r = 0.410, *p* < 0.01 and r = 0.328, *p* < 0.05 respectively). Likewise, the results of the study revealed a moderate relationship between global SOC and one’s attitude toward seeking professional psychological help (r = 0.366, *p* < 0.05). This indicates that the sense of coherence increases an attitude towards seeking help from mental health professionals in those situations when it is really needed.

## 4. Discussion

In this study the relationships between selected demographic and psychological variables and attitude toward seeking help from mental health professionals were scrutinised. At first, the effects of gender was examined. It was expected that men and women would report different intentions to seek psychological help. Consistently with former research [21,22,23], women reported greater willingness to utilize help offered by mental health specialists than men. The difference that exists between women’s and men’s expectations may reflect not only women’s more positive attitude but also their greater openness to discuss personal problems. This finding seems to be in accordance with past research: women displayed more positive attitudes toward mental health counselling and [18,24,25,26,27], and this included varying cultural settings [3,28]. The gender differences in attitudes observed in this study could be party explained by different patterns of socialisation. According to the traditional model of masculinity, widely accepted and well-rooted in Poland, men should solve their problems by themselves, otherwise they might be seen as dependent and weak. Furthermore, men may feel certain reservation toward displaying psychological problems, and thus they tend to have lower motivation to seek consultation and therapy. This observation was earlier supported by a number of previous studies [23,29]. The results of the correlation analysis showed that there is a positive relationship between attitudes toward psychological help-seeking and graduate level of university education. This finding indicates that the level of education helps to predict in some way one’s attitude toward seeking help from mental health professionals, and this is essentially in accordance with previous studies carried out in this field [26,30]. However, there are other studies that show that the level of education is less significant in accounting for those factors that influence one’s attitude toward seeking professional psychological help [31].The analysis of the role of the social background revealed no significant differences between participants from urban and rural area in the attitudes studied here. One may conclude that the socio-cultural differences between these two backgrounds have apparently been diminishing in Poland in recent decades and, as a result of this, people are more likely to employ similar health belief system. However, due to the unquestionable importance of this factor found in previous investigations, future research will contribute to providing the full picture of the role of this variable in determining attitude toward help-seeking attitude.

Likewise, our findings have also revealed significant relationship between health locus of control and the attitude toward seeking professional psychological help. Specifically, the results suggest that internally oriented young adults showed more positive attitude than those externally oriented. This observation is consistent with the results found in other studies which suggest that “internals” are stronger motivated to engage in all types of preventive behaviours enhancing their mental health [8,9]. At the same time, one may speak of a significant positive correlation between sense of coherence and help-seeking attitude, and this relates both to the global SOC scores and its three components. Among them, the most important role seems to be played by comprehensibility. This finding could be interpreted in terms of joint contribution of all three elements of the SOC. This also means that, in order to understand attitudes of young adults better, researchers must necessarily consider a clustering influence of personality dispositions. Moreover, it also implies that the picture of the body of factors that determine attitudes toward seeking help from mental health professionals is much more complex than the variables put to work in this research task. Polish young adults showed explicit confidence in mental health providers and tend to see professional mental health services less stigmatised that could be expected. Nevertheless, one of the limitations of this study is that the research sample comprised a specific stratum of the general population, therefore any hard-and-fast generalisation of obtained results is of limited value and should be verified on other research subject.

## 5. Conclusions

Based on the findings of this research task, attitudes toward psychological help-seeking are significantly related to female gender, graduate university education, internal health locus of control and sense of coherence.

Further research on the issue is necessary and required in Poland in order to validate these results obtained in different age and social groups.

This research is important in terms of practice, and it may be beneficial for mental health professionals to evaluate their clients’ distorted expectations and inadequate attitudes which may greatly affect the therapeutic and counselling processes.

## Figures and Tables

**Table 1:** Socio-demographic characteristics of the sample.

Variables	N	%
Gender		
Males	622	36.64
Females	1084	63.54
Level of university education		
Undergraduate studies	943	55.27
Graduate studies	763	44.73
Social background		
Urban	715	41.91
Rural	991	58.09

**Table 2:** Comparison of the ATSPPHS results by variables.

Variables	N	Mean%	SD	*t*	*p*
Gender					
Males	622	21.12	3.55	2.75	0.01
Females	1084	24.85	4.16		
Level of University Education					
Undergraduate Studies	943	20.46	3.34	2.92	0.005
Graduate Studies	763	25.70	4.51		
Social Background					
Urban	715	23.18	4.27	1.27	NS
Rural	991	21.54	3.66		

Notes: SD—standard deviation, NS—not significant.

**Table 3:** Comparison of the MHLC results by variables.

Locus of Control	Males (*N* = 622)		Females (*N* = 1084)		*t*	*p*
	Mean	SD	Mean	SD		
Internal (I)	28.72	5.64	25.36	4.83	2.17	0.05
Influence of others (O)	17.30	4.81	21.84	5.11	2.84	0.01
Chance (C)	18.45	4.57	17.26	4.66	1.13	NS
**Health Locus of Control**	**Undergraduate (*N* = 943)**		**Graduate (*N* = 763)**		***t***	***p***
	**Mean**	**SD**	**Mean**	**SD**		
Internal (I)	23.60	4.35	29.72	5.34	2.96	0.01
Influence of others (O)	19.08	5.17	18.20	4.78	0.88	NS
Chance (C)	19.85	4.12	15.43	3.52	2.37	0.05
**Locus of Control**	**Urban Area (*N* = 715)**		**Rural Area (*N* = 991)**		***t***	***p***
	**Mean**	**SD**	**Mean**	**SD**		
Internal (I)	27.60	5.37	20.82	5.51	3.08	0.01
Influence of others (O)	18.10	4.94	21.45	4.93	2.15	0.05
Chance (C)	17.88	4.85	19.16	5.02	0.76	NS

Notes: SD—standard deviation, NS—not significant.

**Table 4:** Comparison of the SOC-29 results by variables.

Domains of SOC	Males (*N* = 622)		Females (*N* = 1084)		*t*	*p*
	Mean	SD	Mean	SD		
Meaningfulness	44.31	7.64	45.05	8.13	0.89	NS
Comprehensibility	47.59	8.12	43.21	7.64	1.98	0.05
Manageability	47.42	8.53	41.85	7.48	2.67	0.01
Global SOC	139.32	23.10	131.11	21.35	2.05	0.05
**Domains of SOC**	**Undergraduate (*N* = 943)**		**Graduate (*N* = 763)**		***t***	***p***
	**Mean**	**SD**	**Mean**	**SD**		
Meaningfulness	42.14	8.20	46.82	7.81	2.42	0.01
Comprehensibility	43.71	9.04	47.44	8.26	1.99	0.05
Manageability	44.65	8.42	45.87	7.75	1.18	NS
Global SOC	130.50	22.76	140.13	21.33	2.21	0.05
**Domains of SOC**	**Urban Area (*N* = 715)**		**Rural Area (*N* = 991)**		***t***	***p***
	**Mean**	**SD**	**Mean**	**SD**		
Meaningfulness	45.51	7.94	43.86	9.08	1.09	NS
Comprehensibility	44.14	8.37	44.76	8.25	0.76	NS
Manageability	42.67	8.08	48.35	7.62	2.56	0.01
Global SOC	132.32	22.16	136.89	20.76	1.23	NS

Notes: SD—standard deviation, NS—not significant.

**Table 5:** Pearson correlation between dependent variable (ATSPPH) and independent variables.

Sociodemographic Variables	N	r	Psychological Variables	N	r
Gender			Health locus of control		
Male	622	0.229	Internal (I)	1706	0.536 **
Female	1084	0.345 *	Influence of others (O)	1706	−0.159
Level of education			Chance (C)	1706	−0.089
Undergraduate studies	943	0.268	Domains of SOC		
Graduate studies	763	0.512 **	Meaningfulness	1706	0.328 *
Social background			Comprehensibility	1706	0.473 **
Urban area	715	0.158	Manageability	1706	0.410 **
Rural area	991	0.204	Global SOC	1706	0.366 *

Notes: * *p* < 0.05; ** *p* < 0.01.
